# Impact of Mild Field Drought on the Aroma Profile and Metabolic Pathways of Fresh Tea (*Camellia sinensis*) Leaves Using HS-GC-IMS and HS-SPME-GC-MS

**DOI:** 10.3390/foods13213412

**Published:** 2024-10-26

**Authors:** Xiaohui Liu, Fabao Dong, Yucai Li, Fu Lu, Botao Wang, Taicen Zhou, Degang Zhao, Mingzheng Huang, Feifei Wang

**Affiliations:** 1 Plant Conservation & Breeding Technology Center, Guizhou Key Laboratory of Agricultural Biotechnology, Guizhou Institute of Prataculture, Guizhou Academy of Agricultural Sciences, Guiyang 550006, China; liuxiaohui0908@hotmail.com; 2 Key Laboratory of Plant Resources Conservation and Germplasm Innovation in Mountainous Region (Ministry of Education), College of Tea Sciences, Guizhou University, Guiyang 550025, China; 3 College of Food and Pharmaceutical Engineering, Guizhou Institute of Technology, Guiyang 550025, China; fabao1234@163.com (F.D.); liyucai2860@hotmail.com (Y.L.); 17586454497@163.com (F.L.); 17585632907@163.com (B.W.); a1747632830@163.com (T.Z.); 4 Key Laboratory of Plant Functional Genomics of the Ministry of Education, Jiangsu Key Laboratory of Crop Genomics and Molecular Breeding, Jiangsu Co-Innovation Center for Modern Production Technology of Grain Crops, Institutes of Agricultural Science, Yangzhou University, Yangzhou 225009, China; feifei.wang@yzu.edu.cn

**Keywords:** *Camellia sinensis* cultivar, drought stress, fresh tea leaves, gas chromatography–ion mobility spectrometry (GC-IMS), headspace solid-phase micro-extraction gas chromatography–mass spectrometry (HS-SPME-GC-MS), aroma profile, odor activity values (OAVs), odor-active aroma compounds

## Abstract

Aroma plays a pivotal role in defining tea quality and distinctiveness, and tea producers have often observed that specific drought conditions are closely associated with the formation and accumulation of characteristic aroma compounds in tea leaves. However, there is still limited understanding of the differential strategies employed by various tea cultivars in response to drought stress for the accumulation of key volatile aroma compounds in fresh tea leaves, as well as the associated metabolic pathways involved in aroma formation. In this study, two widely cultivated tea cultivars in China, Fuding Dabai (FD) and Wuniuzao (WNZ), were examined to assess the impact of mild field drought stress on the composition and accumulation of key volatile aroma compounds in fresh leaves using headspace gas chromatography–ion mobility spectrometry (HS-GC-IMS) and headspace solid phase micro-extraction gas chromatography–mass spectrometry (HS-SPME-GC-MS) technologies. Results revealed that drought stress led to a substantial increase in the diversity of volatile compounds (VOCs) in FD, while WNZ exhibited a notable rise in low-threshold VOC concentrations, amplifying sweet, floral, fruity, and earthy aroma profiles in post-drought fresh leaves. Through partial least squares discriminant analysis (PLS-DA) of HS-GC-IMS and HS-SPME-GC-MS data, integrating variable importance projection (VIP) scores and odor activity values (OAVs) above 1, 9, and 13, key odor-active compounds were identified as potential markers distinguishing the drought responses in the two cultivars. These compounds serve as crucial indicators of the aromatic profile shifts induced by drought, providing insights into the differential metabolic strategies of the cultivars. Additionally, KEGG enrichment analysis revealed 12 metabolic pathways, such as terpenoid biosynthesis, fatty acid synthesis, cutin, suberine, and wax biosynthesis, and phenylalanine metabolism, which may play crucial roles in the formation and accumulation of VOCs in tea leaves under drought stress. These findings provide a comprehensive framework for understanding the cultivar-specific mechanisms of aroma formation and accumulation in tea leaves under mild drought conditions.

## 1. Introduction

Tea is a globally popular beverage known for its distinctive flavor and health-promoting properties [1,2,3]. Tea products are primarily derived from the buds and young or mature leaves of the tea plant (*Camellia sinensis*) [4]. Consequently, the bioactive compounds responsible for tea’s flavor and health qualities are predominantly found in these buds and leaves. Several factors, including tea plant cultivar [5,6,7], environmental growing conditions [8,9,10,11,12], and post-harvest processing methods [13,14,15,16], significantly influence the production and accumulation of these active metabolites, thereby affecting the overall quality of the tea. The quality of fresh tea leaves is a fundamental factor in determining the final quality of the processed tea product. It is generally accepted that only high-quality fresh leaves can produce premium tea. The accumulation of bioactive compounds in fresh tea leaves is closely related to both the tea plant cultivar and its growing environment. Among environmental factors, drought is a common stressor that significantly affects tea plant growth and the accumulation of metabolites in young shoots [17,18,19]. Some studies have shown that moderate drought can enhance tea flavor by balancing specific metabolite levels in fresh leaves [19], while slight drought stress has been found to promote polyphenol synthesis, leading to improved tea flavor quality [20]. In addition, drought stress is also known to influence the formation of tea’s unique aromatic compounds. For example, the characteristic aromas of Keemun black tea from China, high-aroma Ceylon tea from Sri Lanka, and Darjeeling tea from India have been associated with short-term drought conditions [21]. In China’s Yunnan province, local tea plantation smallholders also observe that spring drought positively influences the aroma of tea [22]. In a previous study on *Lingtou Dancong* tea plants under drought stress, it was found that different levels of drought can induce the synthesis of varying types of aromatic compounds, with the number of aroma compounds in fresh tea leaves increasing as drought stress intensifies [23]. Additionally, research indicates that slight drought stress can lead to the enrichment of metabolic pathways involved in linoleic acid and butyric acid metabolism, further suggesting drought’s role in modulating tea aroma [20].

Despite the existing research on the positive effects of moderate or slight drought on tea flavor and aroma, the mechanisms by which drought stress specifically influences the formation of key aroma compounds in tea remain insufficiently explored. Volatile compounds (VOCs) in tea, although comprising only 0.01% of the dry weight, are crucial for its aroma [24]. To date, over 700 VOCs have been identified in tea, predominantly alcohols, aldehydes, ketones, and terpenes [4]. However, only a limited number of these compounds, with odor activity values (OAVs) exceeding 1, are considered to make significant contributions to the overall aroma, thus being classified as key odor-active aroma compounds [25,26]. Most previous research has focused on identifying key odor-active aroma compounds in processed tea [13,27,28,29], with limited attention given to fresh tea leaves prior to processing, particularly concerning the effects of abiotic stresses like drought on the formation of key odor-active aroma compounds in fresh tea leaves. This study aims to address this gap by evaluating the effects of field drought stress on the key odor-active aroma compounds in fresh leaves from two widely cultivated tea cultivars in China, Fuding Dabai (FD) and Wuniuzao (WNZ). The primary objective is to assess the impact of drought on the volatile profile and aroma characteristics of fresh tea leaves and to elucidate the differential metabolic pathways involved in aroma formation under drought conditions between the two cultivars.

Gas chromatography–mass spectrometry (GC-MS) is a widely used technique for the identification and quantification of VOCs in tea [4,14,30]. With advances in technology, pre-concentration techniques for VOCs and methods for their separation and identification, centered around GC-MS, have been improved. In particular, headspace solid-phase micro-extraction (HS-SPME) technology offers significant advantages for collecting and concentrating VOCs from tea leaves. This method allows for the efficient extraction of trace aroma compounds in a highly sensitive and rapid manner, enabling ultra-trace (nanogram-level) quantification when combined with GC-MS [13,30,31,32]. Additionally, gas chromatography–ion mobility spectrometry (GC-IMS), a novel technique for the separation, identification, and quantification of VOCs, has also gained prominence in tea research. Compared to HS-SPME-GC-MS, GC-IMS offers the advantage of direct extraction and analysis of volatiles from tea samples without the need for extensive pretreatment, making it particularly effective in separating isomers and complementing HS-SPME-GC-MS in volatile aroma profiling [24,33]. 

In this study, both HS-GC-IMS and HS-SPME-GC-MS were employed to comprehensively analyze the effects of drought stress on the volatile profiles of fresh tea leaves from FD and WNZ cultivars. Key odor-active aroma compounds were identified based on their OAVs, and differences in key aroma markers between the two tea cultivars under drought conditions were assessed using variable importance in projection (VIP) scores in a partial least squares discriminant analysis (PLS-DA) model. Furthermore, KEGG pathway enrichment analysis was performed to investigate the differential metabolic strategies adopted by the two cultivars in response to drought stress. The findings from this study offer valuable insights into how drought stress affects the composition and presentation of key odor-active aroma compounds in fresh tea leaves, particularly via terpenoid biosynthesis, phenylalanine metabolism, fatty acid synthesis, and cutin, suberine, and wax biosynthesis. These results contribute to our understanding of the mechanisms responsible for aroma formation, particularly in cultivar-specific responses to drought stress, while also providing a foundation for optimizing water management in tea cultivation and refining processing techniques to enhance tea quality.

## 2. Materials and Methods

### 2.1. Experimental Design and Collection of Tea Samples 

The experimental tea garden is situated at Hongfengshanyun Tea Farm, Qingzhen, Guizhou Province, China (106°22′ E, 26°31′ N). Eight-year-old ‘Fuding Dabai’ (FD) and ‘Wuniuzao’ (WNZ) tea plants were used as the experimental materials. Tea plants grown under field conditions were subjected to a 20-day natural drought treatment without irrigation, representing the drought-stressed groups (FD-D and WNZ-D, with soil water content at 55% ± 2.5%). In contrast, the control groups (FD-CK and WNZ-CK) were maintained under normal irrigation conditions, with soil water content at 75% ± 2.5%. Tea samples were randomly collected on 26 July 2022, with three biological replicates for each sample. The collected samples, consisting of one bud and two leaves, were promptly frozen in liquid nitrogen and stored at −80 °C in an ultra-low temperature freezer for subsequent aroma analysis.

### 2.2. Chemical Reagents

Aroma internal standard ethyl decanoate (98%) was purchased from TCI (Shanghai, China). Aroma standards of linalool (98%), geraniol (98%), citronellol (95%), phenethyl alcohol (99.10%), 1-octanol (99.5%), citral (95%), decanal (98%), undecanal (97%), hexadecanal (97%), benzaldehyde (99.50%), methyl heptenone (99.50%), ionone (95%), and methyl hexanoate (99.5%) were purchased from Macklin (Shanghai, China). Aroma standards of 2,4-di-tert-butylphenol (98%), (+)-limonene (99%), nerol (98%), and coumarin (98%) were purchased from Yuanye (Shanghai, China). Tea infusion blank matrix standards of epicatechin (EC, 95%), (−)-epigallocatechin gallate (EGCG, 95%), (+)-catechin (C, 95%), and L-theanine (98%) were provided by Macklin (Shanghai, China). Tea infusion blank matrix standards of epigallocatechin (EGC, 98%) and epicatechin gallate (ECG, 98%) were provided by Bidepharm (Shanghai, China). The tea infusion blank matrix standard for caffeine (99.7%) was provided by TMstandard (Beijing, China). Analytical reagents sodium chloride and ethanol absolute were obtained from Jinshan (Chengdu, China). Distilled water was bought from Wahaha Group (Hangzhou, China) and a mixture of n-alkanes (C7–C30) was purchased from Sigma-Aldrich (St. Louis, MO, USA). n-Alkanes (C4–C9), including 2-butanone (99.5%), 2-pentanone (99.5%), 2-hexanone (99.5%), 2-heptanone (98%), 2-octanone (99%), and 2-nonanone (99%), were sourced from Sinopharm (Shanghai, China).

### 2.3. HS-GC-IMS Analysis

The VOCs and polymers of low molecular weight in the sample were detected using the FlavourSpec^®^ (Melbourne, Australia) flavor analyzer, which employs GC-IMS technology. The FlavourSpec^®^ flavor analyzer was equipped with a gas-phase ion mobility spectrometry unit and an automatic headspace sampling unit. The sample preparation procedure was as follows: 1 g of the sample was weighed and placed in a 20 mL headspace vial, followed by the addition of 20 μL of the internal standard ethyl decanoate (100 ppm) and mixing. The sample was then incubated at 60 °C for 15 min before injecting 500 μL of the headspace. The incubation speed was set to 500 rpm, and the headspace injection needle temperature was maintained at 85 °C. Each sample was analyzed in triplicate to ensure reproducibility.

The gas chromatography conditions were as follows: an MXT-5 column (15 m × 0.53 mm × 1.0 μm, Restek, Bellefonte, PA, USA) was used. The column temperature was maintained at 60 °C, with nitrogen (purity 99.999%) as the carrier gas. The total chromatographic run time was 20 min, with the carrier gas flow rate gradient set to an initial flow rate of 2 mL/min maintained for 2 min, and then linearly increased to 100 mL/min over the remaining 18 min. For ion mobility spectrometry, the drift tube temperature was set at 45 °C, and nitrogen (purity ≥99.999%) was used as the drift gas, with a flow rate of 150 mL/min.

The qualitative analysis of volatile substances was conducted using the VOCal software integrated with the National Institute of Standards and Technology (NIST), Flavors and Fragrances of Natural and Synthetic Compounds (FFNSC) library, and the IMS database from G.A.S. (Dortmund, Germany). Retention index (RI) values of the target VOCs were calculated using n-alkanes (C4–C9) as external standards. The identification of VOCs was achieved by matching RI and drift time (Dt) against the NIST and IMS database. The internal standard method was used for quantitative analysis. 

### 2.4. HS-SPME-GC-MS Analysis

HS-SPME-GC-MS provides significant advantages in the qualitative and quantitative analysis of VOCs in complex samples, such as superior enrichment, heightened sensitivity, and enhanced selectivity. This technique is particularly effective for the dissociative qualitative analysis of high-molecular-weight VOCs. Consequently, the Shimadzu GCMS-TQ8040 NX triple quadrupole GC-MS system (Shimadzu, Kyoto, Japan), combined with HS-SPME, was utilized for the analysis of VOCs in tea leaf samples. The gas chromatographic separation was conducted using an InertCap 5MS capillary column (30 m × 0.25 mm, 0.25 μm, Shimadzu, Kyoto, Japan), with the AOC-6000 Plus Multifunctional Autosampler (Shimadzu, Kyoto, Japan) facilitating fully automated sample injection. The extraction and analysis of VOCs were performed following the published experimental protocol, with certain modifications [34,35]. 

#### 2.4.1. Sample Preparation

An appropriate amount of tea leaf sample was placed in a mortar and ground with liquid nitrogen. Precisely 0.5 g of the ground sample was then transferred into a 20 mL headspace vial, followed by the addition of 1.5 g of NaCl and 5 mL of boiled ultrapure water. Additionally, 4 μL of ethyl decanoate (1 mg/L, diluted in anhydrous ethanol) was incorporated as an internal standard. The vial was promptly sealed with a screw cap fitted with a PTFE–silicon spacer and positioned in the AOC-6000 Plus (Shimadzu, Kyoto, Japan) sample tray in preparation for detection. 

#### 2.4.2. HS-SPME Extraction of VOCs

The HS-SPME procedure was configured as follows: the sample was equilibrated at 70 °C for 5 min, after which the SPME fiber (50/30 μm DVB/CAR/PDMS, CTC Analytics, Zwingen, Switzerland) was exposed to the headspace for extraction at 70 °C for 30 min, with the agitator set to 600 rpm. The fiber was then inserted into the GC injection port in splitless mode for desorption for 5 min, followed by a post-conditioning time of 5 min.

#### 2.4.3. GC-MS Analysis of VOCs

The separation of VOCs was performed using an InertCap 5MS capillary column (30 m × 0.25 mm, 0.25 μm) with helium (purity >99.999%) as the carrier gas. The GC program was set as follows: an initial temperature of 40 °C was maintained for 3 min, followed by a ramp of 2 °C/min to 190 °C, held for 1 min, and then a second ramp of 10 °C/min to 230°C, held for 2 min. The flow rate of the carrier gas was set to 1 mL/min, and the column oven cooling rate was adjusted to medium. The MS conditions were as follows: an electron impact (EI) ion source was operated at an electron energy of 70 eV. The ion source and interface temperatures were both set to 280 °C. A solvent delay time of 2 min was applied, and the mass scanning range was established from m/z 33 to 600. Each sample was subjected to triplicate injections for analysis.

#### 2.4.4. Identification and Quantification of VOCs

All VOCs were initially identified using the NIST 17 and FFNSC1.3 library match, selecting aroma compounds with a similarity score exceeding 80%. These VOCs were further confirmed through their RI and authentic standards. n-Alkane (C7-C30) standards were utilized to determine the RI of VOCs. Each aroma standard compound, at a specified concentration, was mixed with the ‘volatile-free’ blank matrix and subjected to qualitative and quantitative analysis via HS-SPME-GC-MS to obtain calibration curves. For compounds lacking available standards, estimation was conducted using standards with analogous functional groups and comparable carbon atom counts [34,35]. The standard curve method was employed for the absolute quantitative analysis of certain VOCs, while the internal standard method was utilized for the semi-quantitative analysis of other VOCs. The details of the ‘volatile-free’ blank matrix model stock solution and the calibration standard curves are shown in Tables S1 and S2.

#### 2.4.5. Odor Activity Value (OAV) Analysis of VOCs 

OAV is a critical indicator for determining key aroma-active compounds in a sample. The OAV is calculated as the ratio of the concentration of each volatile compound to its odor threshold (OT) in water, with the thresholds primarily referenced from early relevant studies [4,36,37,38]. When multiple data points are available for the threshold, the selection criteria prioritize using data from aqueous systems and the most recent data. Generally, when the OAV exceeds 1, the compound significantly influences the tea’s overall flavor. With an OAV between 0.1 and 1, the compound acts as a modifying aroma, subtly enhancing the flavor profile [39]. The higher the OAV, the greater the compound’s contribution to the overall flavor, and vice versa. 

### 2.5. Data Analysis 

All experiments were performed in triplicate and the corresponding results are presented as the mean ± standard deviation (SD). Statistical analysis of VOC differences among the groups was conducted using one-way analysis of variance (ANOVA) with SPSS software 26 (IBM, Armonk, NY, USA), and *p*-values < 0.05 were considered statistically significant. Multivariate statistical analysis of the experimental data was performed using MetaboAnalyst 6.0 (https://www.metaboanalyst.ca/, accessed on 7 September 2024) and SIMCA 14.1 (Umetrics, Umea, Sweden), focusing on Principal Component Analysis (PCA) and partial least squares discriminant analysis (PLS-DA) to analyze the similarities and differences in VOCs across different samples. The variable importance in projection (VIP) method, derived from PLS-DA models, was utilized to identify the key aroma compounds responsible for distinguishing between different tea cultivars and the effects of drought treatments. The alterations in VOCs following drought treatment were illustrated using a volcano plot. Significant changes were defined by a *p*-value < 0.05 (*t*-test) and a fold change (FC) of ≥2 or ≤0.5. The volcano plot was generated with MetaboAnalyst 6.0. Graphical representations of the data were created using OriginPro 2021 (OriginLab, Northampton, MA, USA), MetaboAnalyst 6.0, Reporter and Gallery Plot plug (G.A.S., Dortmund, Germany), and the Biodeep cloud platform from Panomix (Suzhou, China). 

## 3. Results and Discussion

### 3.1. Qualitative and Quantitative Analysis of the VOCs by HS-GC-IMS

**Figure 1:**
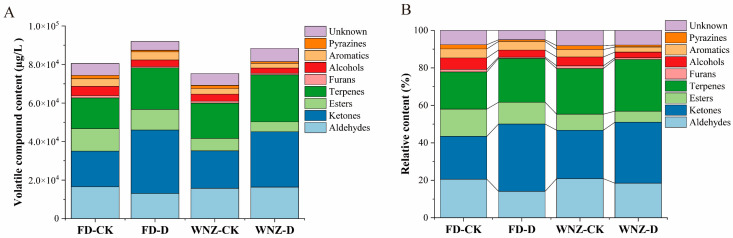
Comparison of VOCs in 4 groups detected by HS -GC-IMS. (**A**) Stacked column graph of VOC content; (**B**) 100% stacked column graph of VOC relative content.

Aroma volatiles of fresh tea leaves were identified and analyzed through HS-GC-IMS, employing retention indexes (RIs), retention times, and drift times referenced from the NIST library. A total of 50 peaks were detected across four groups, with 44 peaks successfully identified as specific chemical compounds, while 6 peaks remained unidentified and were classified as unknown substances, requiring further validation. The detailed analysis results are provided in Table S3. The detected VOCs were categorized into several groups, including aldehydes, ketones, esters, terpenes, furans, alcohols, aromatics, and pyrazines (Figure 1A). Among these, aldehydes, ketones, and terpenes were the three most dominant volatile groups, collectively constituting over 60% of the total volatiles (Figure 1B), with 12, 7, and 6 aroma compounds, respectively (Table S3).

The VOC content exhibited variation between different tea cultivars. Overall, the FD cultivar demonstrated higher VOC levels compared to the WNZ cultivar. Drought stress led to an increase in VOC levels in both cultivars. Notably, in the FD cultivar, ketones, terpenes, and aromatics showed a substantial increase under drought conditions, while aldehydes, esters, furans, alcohols, and pyrazines decreased. In comparison, in the WNZ cultivar, aldehydes, ketones, and terpenes exhibited a marked increase under drought stress, while the levels of esters, furans, alcohols, aromatics, and pyrazines decreased. The results indicate that the differential accumulation of aroma compounds under drought stress is related to the varying drought adaptability of different tea cultivars [40].

In summary, across both cultivars, drought stress induced a notable increase in ketones and terpenes, contributing to enhanced citrus, floral, sweet, earthy, and herbal aroma characteristics in fresh tea leaves. These results suggest that ketones and terpenes play a key role not only in the drought resilience of tea plants but also in the modulation of tea flavor under environmental stress. Interestingly, tea plantation smallholders serving as informants from tea production communities in Yunnan Province, China, also observed that the aroma of spring tea becomes more intense during drought periods, which they believed positively influences the market price of the tea [22]. This observation aligns with the findings of our study, where the content of volatile compounds increased in both FD and WNZ tea cultivars following drought stress (shown in Figure 1A).

**Figure 2:**
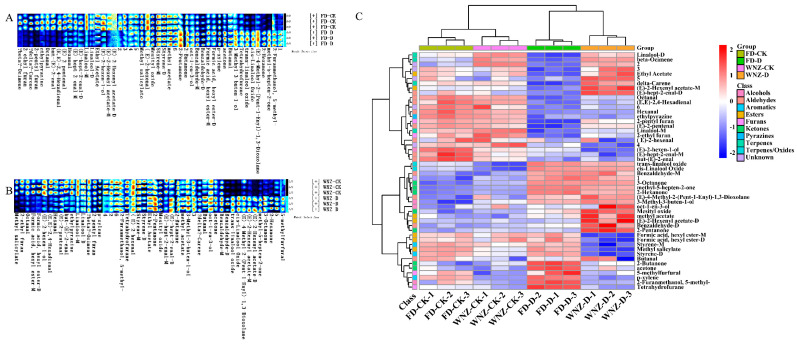
Comparisons of the identified VOCs in 4 groups by HS-GC-IMS. (**A**) The VOC fingerprint comparisons of FD-CK and FD-D; (**B**) the VOC fingerprint comparisons of WNZ-CK and WNZ-D; (**C**) heatmap clustering of VOCs in FD-CK, FD-D, WNZ-CK, and WNZ-D.

Drought stress influences the differential accumulation of VOCs in the fresh leaves of tea plants. Compared to the control group, drought-stressed plants exhibited higher levels of ketone VOCs, including methyl-5-hepten-2-one, 3-octanone, mesityl oxide, 2-pentanone, 2-butanone, and acetone. Additionally, methylated VOCs ((E)-4-methyl-2-(pent-1-enyl)-1,3-dioxolane, methyl acetate, 5-methylfurfural, 2-furanmethanol, 5-methyl-, and 3-methyl-3-buten-1-ol), linalool oxides (trans-linalool oxide, cis-linalool oxide), (E)-2-hexenyl acetate-D, oct-1-en-3-ol, p-xylene, and benzaldehyde were also elevated (Figure 2A–C). These VOCs contribute predominantly to sweet, caramel-like, floral, earthy, and fruity aroma characteristics (shown in Table S3). 

It is important to note that certain VOCs exhibited distinct accumulation patterns across different tea cultivars. For example, VOCs such as (E)-hept-2-enal-D, butanal, (E)-2-hexenyl acetate-M, ethyl acetate, and delta-carene were present in significantly higher concentrations in the WNZ cultivar under drought stress (Figure 2B), whereas their levels decreased in the FD cultivar under the same conditions (Figure 2A). Conversely, formic acid, hexyl ester-M, formic acid, hexyl ester-D, and styrene-D concentrations were elevated in cultivar FD following drought stress but decreased in WNZ (Figure 2A,B). These variations in VOC accumulation between cultivars are likely linked to differences in their metabolic adaptations to drought stress.

Moreover, an intriguing finding from this study was the increase in methylated volatile compounds under drought stress, which contributed to the enhanced sweet and fruity characteristics of fresh tea leaves. However, due to the absence of molecular and epigenetic data, the specific mechanisms by which drought conditions regulate the biosynthesis of methylated VOCs in tea remain unclear. Previous studies have suggested that environmental stresses, such as cold and drought, as well as postharvest processing, can induce changes in 5-methylcytosine (5mC) DNA methylation [17,41,42]. These epigenetic modifications are known to affect the expression of key genes involved in flavor biosynthesis, subsequently influencing the accumulation of compounds such as theanine, catechins, terpenoid [41], and indoles [42]. Investigating DNA methylation as a potential regulator of increased methylated VOCs in response to drought stress in tea could serve as a promising avenue for future research. 

It is noteworthy that the alteration in linalool oxide levels observed under drought stress may be indicative of its role as a signaling molecule in the tea plant’s stress response. Previous research has demonstrated that linalool can be converted to linalool oxides via 6,7-epoxylinalool, and that infestations by green leafhoppers activate the metabolic flux from linalool to linalool oxides and their glycosides [43]. These findings may shed light on the regulatory mechanisms underlying linalool oxide accumulation in response to stress in tea plants. 

In conclusion, the present study identifies several VOCs as potential metabolic markers for breeding drought-resistant tea cultivars with enhanced aroma profiles. These insights may also provide a basis for improving tea quality through targeted manipulation of metabolic pathways involved in aroma compound biosynthesis.

### 3.2. Identification of VOCs by HS-SPME-GC-MS

**Figure 3:**
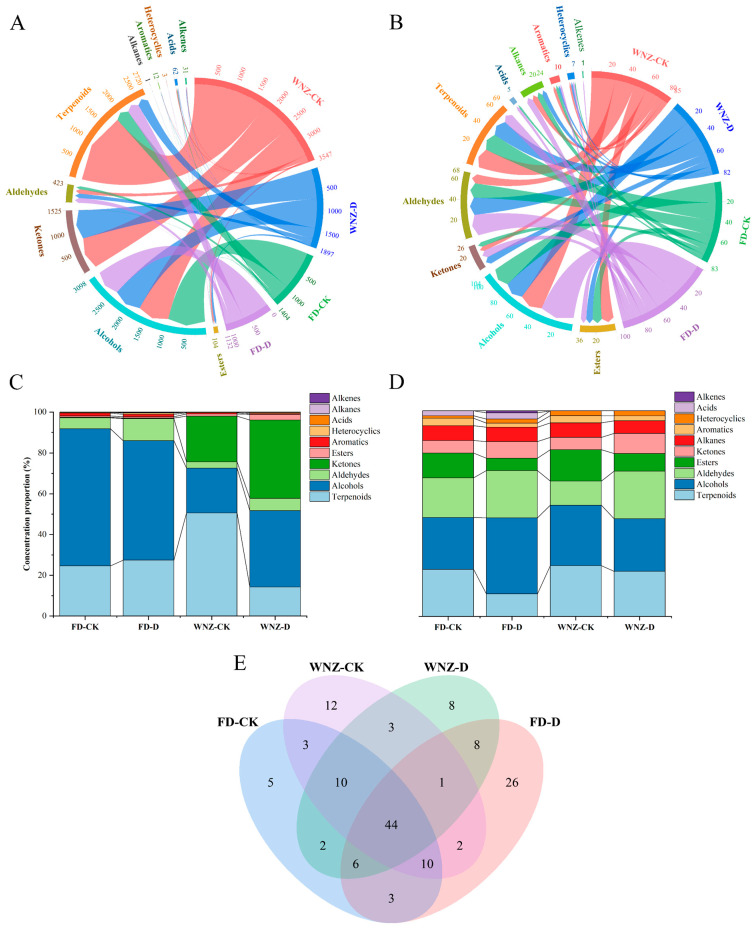
Volatile profiles across the four groups detected by HS-SPME-GC-MS. (**A**) Chord plot of aggregate VOC concentrations by category; (**B**) chord plot of total VOC numbers in each category; (**C**) 100% stacked graph of concentration proportions for different VOC categories; (**D**) 100% stacked graph of number proportions for different VOC categories; and (**E**) Venn diagram depicting VOC differences across the four groups.

The volatile organic compounds (VOCs) from four tea sample groups were identified using HS-SPME-GC-MS, revealing a total of 144 distinct VOCs. The number of volatiles detected varied across the groups, with 85, 82, 83, and 100 volatiles identified in WNZ-CK, WNZ-D, FD-CK, and FD-D, respectively. These identified volatiles were categorized into 10 classes, namely esters, alcohols, ketones, aldehydes, terpenoids, acids, alkanes, alkenes, aromatics, and heterocyclics (as detailed in Table S4 and Figure 3B). To provide a clearer visualization of the variations in and characteristics of VOCs between groups, the total content, number, and proportion of VOCs in each category were summarized and presented through chord graphs, bar plots, and Venn diagrams (Figure 3). In terms of total VOC content, WNZ samples exhibited higher levels compared to FD samples (Figure 3A). The primary VOC classes in WNZ-CK and WNZ-D were terpenoids, alcohols, and ketones, accounting for 95% and 89% of the total concentration, respectively (Figure 3C). In contrast, FD-CK and FD-D were dominated by alcohols, terpenoids, and aldehydes, with these three categories comprising 97% of the total concentration (Figure 3D).

Following drought stress, a decrease in VOC concentration was observed in both the FD and WNZ cultivars (Figure 3A). Regarding VOC diversity, FD exhibited a greater increase in component richness after drought stress, whereas the variation in WNZ was less pronounced (Figure 3B). In both cultivars, terpenoids, alcohols, ketones, aldehydes, and esters were the predominant VOC classes, collectively contributing 85%, 85%, 87%, and 90% in FD-CK, FD-D, WNZ-CK, and WNZ-D, respectively. In the FD cultivar, drought stress led to an increase in the number of alcohols, aldehydes, and ketones, while the numbers of terpenoids and esters decreased. In comparison, the WNZ cultivar experienced a rise in aldehydes and ketones, but a reduction in alcohols, terpenoids, and esters under drought conditions (Figure 3D). 

Furthermore, 44 volatiles were found to be common across all four groups, while 5, 26, 12, and 8 volatiles were exclusively detected in FD-CK, FD-D, WNZ-CK, and WNZ-D, respectively (Figure 3E). It is noteworthy that while the total VOC content in WNZ-CK exceeded that in FD-CK, the difference in VOC variety between these groups was marginal. Under drought stress, although both cultivars showed a reduction in total VOC content, FD-D exhibited a smaller decrease, along with a notable increase in component richness. By contrast, WNZ-D displayed only a slight reduction in VOC variety but experienced a more significant decline in total VOC content.

These findings are not entirely congruent with those obtained through HS-GC-IMS, likely due to differences in the target compounds and detection principles of the two methodologies. Therefore, employing a complementary, multi-technique approach is essential for achieving a more comprehensive and nuanced characterization of sample attributes [31,44]. 

### 3.3. OAV Analysis of HS-SPME-GC–MS Data 

The contribution of VOCs to the overall aroma profile of tea is influenced not only by their concentrations but also by their respective odor thresholds. The ratio between an odorant’s concentration and its detection threshold, commonly referred to as the odor activity value (OAV), serves as a critical determinant of its impact on the sample’s aroma [4,26,28]. Comprehensive details regarding the concentration, odor threshold, odor descriptions, and OAVs of VOCs across the four sample groups are presented in Table S4. VOCs with OAVs ≥1, as well as those with OAVs between 0.1 and 1, are selectively highlighted and discussed in Table 1.

In total, 45 VOCs exhibited OAVs ≥0.1, with 27 of these compounds showing OAVs greater than 1 across the four groups. Specifically, 15, 16, 14, and 22 VOCs with OAVs ≥1 were identified in FD-CK, FD-D, WNZ-CK, and WNZ-D, respectively. These findings suggest that drought stress prompted an increase in the number of VOCs with OAVs ≥1 in both cultivars, with WNZ-D experiencing a marked 57.14% rise compared to WNZ-CK.

Notably, seven VOCs shared across all four groups exhibited OAVs ≥1, including 1-octen-3-ol (earthy, mushroom), linalool (floral), geraniol (floral), β-damascone (fruity), (E)-β-ionone (floral), citral (citrus), and β-myrcene (spicy). These volatiles contribute significantly to the floral and fruity aromatic characteristics of tea leaves. Linalool, which had the highest OAV, is ubiquitous in a wide range of teas, including green tea, black tea, oolong tea, white tea, and yellow tea. Its production is predominantly linked to the light fermentation and drying phases of tea processing, where it imparts a fresh floral fragrance. In addition, geraniol, β-damascone, (E)-β-ionone, and β-myrcene are typically more abundant in black and oolong teas, particularly during fermentation, oxidation, and rolling processes. In contrast, 1-octen-3-ol and citral are more frequently detected in green and white teas, especially during the light fermentation and drying stages.

Upon drought stress, 10 key VOCs in the FD cultivar with OAVs exceeding 1 showed a notable increase, including 1-octen-3-ol, 6-methyl-5-hepten-2-one, (E)-α-ionone, β-damascone, (E,E)-2,4-heptadienal, octanal, nonanal, citral, dodecanal, and linalool oxide I. These compounds predominantly contribute to enhanced fruity, citrus, and orange aroma profiles. Conversely, nine volatiles demonstrated a decline in OAVs, such as (Z)-hex-3-en-1-ol, linalool, geraniol, (Z)-jasmone, (E)-β-ionone, (E)-2-nonenal, β-myrcene, δ-cadinene, and cedrol, primarily associated with green, grassy, floral, and spicy aromatic characteristics. In contrast, the WNZ cultivar under drought stress exhibited an increase in 15 volatiles with OAVs exceeding 1, including methyl salicylate, 1-octen-3-ol, 6-methyl-5-hepten-2-one, 1-octen-3-one, (E)-α-ionone, β-damascone, hexanal, (E,E)-2,4-heptadienal, octanal, (E)-2-nonenal, (E)-2-decenal, nona-(2E,4E)-dienal, linalool oxide I, and linalool oxide II, which contribute to heightened minty, earthy, citrus, violet, fruity, fatty, spicy, floral and woody aroma notes. On the other hand, eight VOCs displayed reduced OAVs, including hex-(3Z)-enyl acetate, linalool, geraniol, (Z)-jasmone, (E)-β-ionone, nonanal, β-myrcene, and δ-cadinene, characterized by green, floral, orange, spicy, and woody aromas. In summary, these findings suggest that drought stress predominantly intensified citrus and fruity aromas, while diminishing green and grassy notes. A consistent trend across both cultivars was the elevation of OAVs for aldehyde and ketone VOCs, whereas certain alcohol and terpene VOCs exhibited decreased OAVs. Particularly noteworthy is the observed reduction in linalool’s OAV, accompanied by a corresponding increase in linalool oxide’s OAV, indicating a potential conversion of linalool to its oxides in response to environmental stressors, thus contributing to floral and woody aroma characteristics. This phenomenon aligns with the results derived from HS-GC-IMS analyses. 

Synthesis of the data presented in Figure 3 and Table S4 underscores the distinct adaptive responses of the two tea cultivars to drought stress. The FD cultivar’s response was characterized by an expansion in the number of VOCs, while the WNZ cultivar exhibited a strategy of enhancing the concentrations of low-threshold, aroma-active compounds, thereby augmenting their OAVs and leading to a more pronounced aromatic profile. This differential response between cultivars presents an intriguing avenue for future exploration.

### 3.4. Comparative Analysis of HS-GC-IMS and HS-SPME-GC-MS in Identifying VOCs

#### 3.4.1. Comparative Analysis of VOC Quantities and Categories

**Figure 4:**
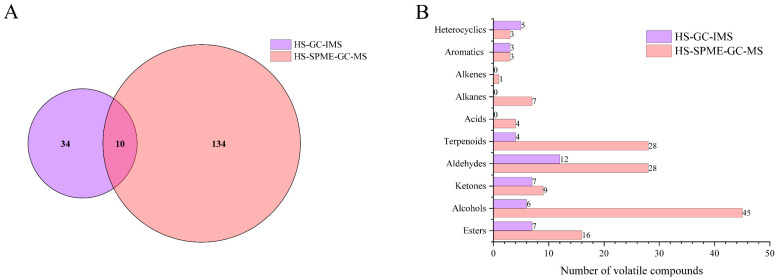
Comparative analysis of VOC quantities and categories: (**A**) Venn diagram of VOC numbers; (**B**) column graph of VOC categories.

As illustrated in Figure 4A, a total of 178 VOCs were identified in this study using two distinct analytical methodologies. Specifically, HS-GC-IMS detected 44 VOCs, while HS-SPME-GC–MS identified 144 VOCs. Among these, 10 volatiles were found to be common across both methods, namely linalool, linalool oxide II, linalool oxide I, 6-methyl-5-hepten-2-one, benzaldehyde, methyl salicylate, octanal, (E)-2-heptenal, 1-octen-3-ol, and hexanal. These shared compounds are integral to the aroma profiles of fresh tea leaves and various tea products [4,24]. 

VOC classification further revealed that HS-GC-IMS grouped the detected volatiles into seven categories, with aldehydes representing the most abundant class. By contrast, the VOCs identified via HS-SPME-GC–MS were categorized into ten groups, with alcohols being the dominant class. Notably, HS-GC-IMS did not to detect acids, alkanes, or alkenes, and the number of VOCs in most other categories, except for heterocyclic and aromatic compounds, was significantly lower compared to those identified by HS-SPME-GC–MS (Figure 4B). This disparity aligns with previous findings in the literature, wherein the two techniques were applied for the analysis of tea aroma compounds. HS-GC-IMS predominantly detects highly volatile and low-boiling-point compounds, whereas HS-SPME-GC–MS is more proficient in enriching and identifying higher-boiling-point volatiles. Moreover, the limited selectivity of IMS, attributed to ion–molecule and ion–ion competition reactions within the ionization chamber [45,46], may further contribute to the lower number of detected volatiles in comparison to HS-SPME-GC–MS.

#### 3.4.2. PCA of All VOCs 

**Figure 5:**
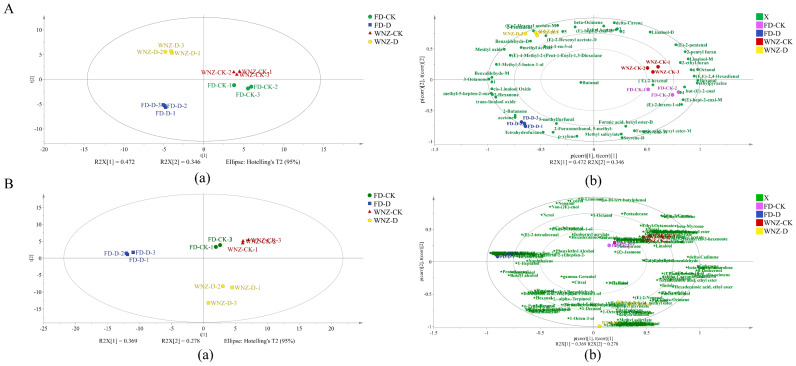
PCA plot and bioplot HS-GC-IMS and HS-SPME-GC-MS. (**A**) PCA (**a**) and biplot (**b**) of VOC intensity detected by HS-GC-IMS; (**B**) PCA (**a**) and biplot (**b**) of VOC concentration detected by HS-SPME-GC–MS.

In order to comprehensively evaluate the discriminative capacity of the two analytical methods applied to the four sample groups, as well as to elucidate the VOCs most strongly correlated with each group, unsupervised PCA analyses were conducted on both datasets, as depicted in Figure 5A,B. Figure 5A illustrates the PCA score plot (Figure 5A(a)) and the corresponding bi-plot (Figure 5A(b)) based on VOC concentration data obtained via HS-GC-IMS. The first two principal components account for 81.8% of the total variance, effectively segregating the four sample groups into distinct quadrants. Control groups are primarily located along the positive axis of PC1, whereas drought-stressed groups occupy the negative axis of PC1. Similarly, WNZ cultivars are positioned on the positive side of PC2, and FD cultivars on the negative side. The above results indicate that both tea cultivars experienced drought stress-induced significant variations in volatile profiles.

Furthermore, Figure 5A(b) identifies several key VOCs strongly associated with WNZ-CK, including 2-pentyl furan (fruity and green), linalool-M (citrus and floral), 2-ethyl furan (Beany, ethereal, and cocoa), (E)-2-pentenal (green and fruity), (E,E)-2,4-Hexadienal (sweet and green), and octanal (waxy and citrus). Collectively, these compounds contribute to the characteristic green, fruity, and citrus aroma of WNZ-CK. In contrast, WNZ-D, situated in the second quadrant, is closely associated with 2-pentanone (sweet, and fruity), (E)-2-hexenyl acetate-D (sweet privet), benzaldehyde-D (bitter, almond, cherry, and burnt sugar), (E)-2-hexenyl acetate-M (sweet privet), oct-1-en-3-ol (mushroom, earthy, and green), and methyl acetate (sweet and fruity), contributing sweet, fruity, and earthy notes to the aroma profile of WNZ-D.

In parallel, FD-D exhibits a strong correlation with VOCs such as 2-butanone (fruity, and camphoraceous), acetone (apple and pear), tetrahydrofuran, 5-methylfurfural (sweet and caramellic), and 2-furanmethanol, 5-methyl- (sweet and caramellic), which collectively imbue FD-D with distinctive fruity, sweet, and caramel-like aromas. On the other hand, FD-CK is highly correlated with (E)-2-hexenal (leafy, green), ethylpyrazine (nutty and coffee), (E)-2-enal, (E)-hept-2-enal-M (green, vegetable), and (E)-2-hexen-1-ol (fresh green and leafy), lending a predominantly leafy, green, and nutty aromatic profile to FD-CK.

Figure 5B illustrates the PCA of VOCs in the four sample groups, as determined by HS-SPME-GC-MS. PC1 and PC2 explain 36.9% and 27.8% of the total variance, respectively. The sample groups are distributed across three quadrants, demonstrating clear discrimination between the control and drought-stressed groups. FD-CK and WNZ-CK are both situated in the first quadrant, while FD-D occupies the second quadrant, and WNZ-D the fourth quadrant (Figure 5B(a)). Notably, PC1 effectively separates the control and treatment groups of the FD cultivar, while PC2 discriminates between the control and treatment groups of the WNZ cultivar. Although FD-CK and WNZ-CK both reside within the first quadrant, their confidence ellipses differ, with FD-CK falling within the 50% confidence interval and WNZ-CK within the 75% confidence interval.

Compared to the HS-GC-IMS results, the HS-SPME-GC-MS analysis reveals a broader range of VOCs (Figure 5B(b)). The results from Figure S1 and Figure 5(Bb) illustrate that VOCs highly correlated with the control groups (WNZ-CK and FD-CK) are predominantly linked to floral, herbal, green, and fruity aroma characteristics. Notable compounds include (Z)-jasmone (floral), theaspirane (tea herbal), delta-elemene (herbal), hexyl isobutyrate (green), linalool oxide IV (tea-like, woody, and floral), undecanal (floral), (Z)-2-hepten-1-ol (fatty), (Z)-hex-3-en-1-ol (green), and nonanoic acid (waxy). In contrast, linalool (floral), (E)-beta-farnesene (herbal), neral (citrus), 1-nonanol (floral), cyclohexanol (green and rummy), alpha-calacorene (woody), (E)-3-hexen-1-ol (green), hex-(3Z)-enyl butyrate (green), acetic acid hexyl ester (fruity), T-cadinol (balsamic), and (Z)-3-hexenyl hexanoate (green) show a strong association with FD-CK.

On the other hand, VOCs significantly correlated with FD-D include caryophyllene oxide (woody), pinocarveol (herbal and woody), perilla alcohol (green, cumin, and spicy), dodecanal (soapy, waxy, and citrus), 1-tetradecanol (waxy), Z-5-decen-1-ol (waxy, fatty, and rose), 10-undecen-1-ol (citrus), dodecanoic acid (fatty and coconut), exo-Isocitral (lemongrass), tetradecanal (waxy, citrus, and fatty), (Z)-9-octadecen-1-ol (fatty), dehydrolinalool (floral), 1-undecanol (fresh, waxy, and rose), 6-hepten-1-ol (herbal), 2-undecenal (fruity), and tridecanal (citrus and grapefruit peel). Conversely, VOCs closely linked to WNZ-D predominantly include linalool oxide I (woody and floral), linalool oxide II (woody and floral), linalool oxide III (woody, floral, and citrus), alpha-farnesene (woody, and citrus), oct-(2E)-enal (fatty), nona-(2E,4E)-dienal (fatty, melon, and waxy), (3Z)-beta-ocimene (floral), (E)-2-decenal (waxy, fatty, and earthy), methyl salicylate (minty), alpha-guaiene (sweet and woody), gamma-muurolene (woody), beta-damascone (fruity and floral), dehydrolinalool (floral), hexadecanoic acid methyl ester (waxy), and 1-octen-3-one (earthy and mushroom). A holistic analysis of these findings indicates that the drought-treated groups (FD-D and WNZ-D) are more closely associated with woody, floral, rose, waxy, fatty, fruity, citrus, and earthy VOCs. Notably, the drought-treated groups exhibit a wider diversity of VOCs, leading to more complex and enriched aroma profiles. One particularly interesting observation is that VOCs correlated with the oxidation and dehydrogenation products of linalool are more prevalent in WNZ-D, primarily in the form of four distinct compounds: linalool oxide I, linalool oxide II, linalool oxide III, and dehydrolinalool. These compounds significantly enhance the floral and woody aroma characteristics in WNZ-D.

#### 3.4.3. Identification of Aroma Markers Based on HS-GC-IMS and HS-SPME-GC–MS Data

In the PCA model, sample data visualization is enhanced by projecting the observations into a reduced dimensional space that captures the variance in the first few principal components, thereby highlighting classification trends across the samples. In contrast, the supervised PLS-DA model seeks to maximize the discrimination between different groups by focusing on the most influential differential metabolites across samples [47]. Specifically, VOCs with VIP > 1 were selected as the key discriminatory features in the PLS-DA model [48], as these VOCs substantially contribute to the overall aroma profile.

**Figure 6:**
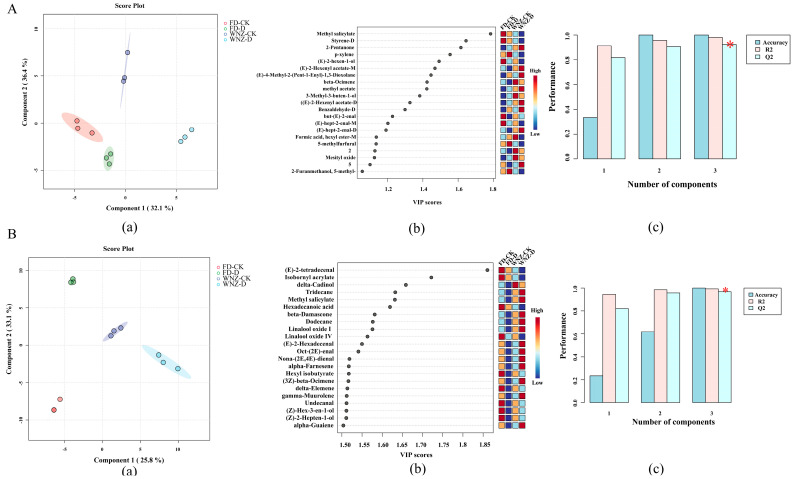
PLS-DA analysis on data obtained from HS-GC-IMS and HS-SPME-GC-MS. (**A**) PLS-DA score plot (**a**), VIP score plot (**b**), and model prediction accuracy for HS-GC-IMS data (**c**); (**B**) PLS-DA score plot (**a**), VIP score plot (**b**), and model prediction accuracy (**c**) for HS-SPME-GC-MS data. Filled circles in panel (a) represent the 95% confidence region and the red star in panel (c) represents the best classifier.

Figure 6 illustrates the effectiveness of PLS-DA modeling in distinguishing the VOC content differences detected via HS-GC-IMS and HS-SPME-GC-MS, alongside the critical aroma volatiles that contribute prominently to sample discrimination. The PLS-DA score plots derived from HS-GC-IMS (Figure 6(Aa)) and HS-SPME-GC-MS (Figure 6(Ba)) datasets explain 68.5% and 58.9% of the total variance, respectively. Both score plots exhibit clear separation of the sample groups, demonstrating the capability of PLS-DA to effectively distinguish between the different sample groups. To validate the accuracy and robustness of the PLS-DA models, cross-validation (CV) was performed, and the statistical parameters—accuracy, R^2^, and Q^2^—all exceeded 0.95 (Figure 6A(c),B(c)), confirming the reliability of the PLS-DA models employed.

Furthermore, in the VIP score plot (Figure 6A(b)), 21 VOCs with VIP > 1 were identified as potential characteristic compounds in the PLS-DA model based on HS-GC-IMS data. Given that aroma presentation is ultimately related to the OAV of VOCs, nine volatiles—including methyl salicylate, Styrene-D, 2-pentanone, (E)-2-hexen-1-ol, β-ocimene, 3-Methyl-3-buten-1-ol, but-(E)-2-enal, (E)-hept-2-enal-M and (E)-hept-2-enal-D—had OAVs all >1, indicating their potential as key aroma markers that drive the discrimination in the HS-GC-IMS PLS-DA model. In comparison, for the PLS-DA model based on HS-SPME-GC-MS data, a total of 52 VOCs had VIP > 1, with 22 VOCs exhibiting VIP > 1.5 (Table S5). Thus, a VIP score plot was generated for these 22 VOCs (Figure 6(Bb)), which could serve as potential characteristic VOCs for the PLS-DA model based on HS-SPME-GC-MS data. Combining the criteria of VIP > 1 and OAV > 1, 13 VOCs—methyl salicylate, β-damascone, linalool oxide I, oct-(2E)-enal, nona-(2E,4E)-dienal, (Z)-hex-3-en-1-ol, (E,E)-2,4-heptadienal, linalool oxide II, cedrol, nonanal, (Z)-jasmone, hexanal, and 1-octen-3-one—were highlighted as critical aroma markers for the HS-SPME-GC-MS-based PLS-DA model.

#### 3.4.4. VOC Change of Different Tea Cultivars Following Drought Stress

**Figure 7:**
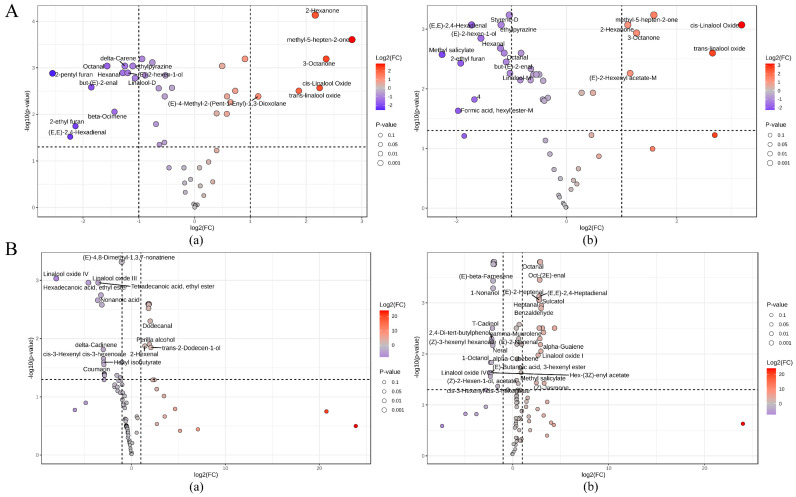
Volcanic map of differential VOCs detected by HS-GC-IMS (**A**) and HS-SPME-GC-MS (**B**). (**a**) FD-D vs. FD-CK; (**b**) WNZ-D vs. WNZ-CK.

To gain deeper insights into the effects of field drought on the VOCs profiles of fresh tea leaves from different cultivars, a within-group comparative analysis was conducted between control and drought-treated samples of the FD and WNZ cultivars, respectively. Specifically, differential VOCs were screened using T-test analysis (*p*-value < 0.05) and fold change (FC ≥ 2 or ≤ 0.5) as the selection criteria. As illustrated in Figure 7, the volcano plots clearly depict the upregulation and downregulation of VOCs between drought-stressed and control samples.

A total of 17 and 18 differential VOCs were identified between the FD-D vs. FD-CK group and the WNZ-D vs. WNZ-CK group using HS-GC-IMS data (Figure 7A). In the FD-D vs. FD-CK comparison, six VOCs were significantly increased, including 2-hexanone, methyl-5-hepten-2-one, 3-octanone, cis-linalool oxide, trans-linalool oxide, and (E)-4-methyl-2-(pent-1-enyl)-1,3-dioxolane. Meanwhile, 11 VOCs were significantly decreased, namely octanal, delta-carene, ethylpyrazine, hexanal, (E)-2-hexen-1-ol, 2-pentyl furan, linalool-D, but-(E)-2-enal, β-ocimene, 2-ethyl furan, and (E,E)-2,4-hexadienal. In contrast, the WNZ cultivar exhibited significant alterations in VOCs under drought stress, with six VOCs showing marked upregulation. These included methyl-5-hepten-2-one, cis-linalool oxide, 2-hexanone, 3-octanone, trans-linalool oxide, and (E)-2-hexenyl acetate-M. Meanwhile, 12 VOCs were significantly decreased, such as styrene-D, (E,E)-2,4-hexadienal, ethylpyrazine, (E)-2-hexen-1-ol, hexanal, octanal, methyl salicylate, but-(E)-2-enal, 2-ethyl furan, linalool-M, an unidentified volatile compound, and formic acid hexyl ester-M. From the above analysis, it is evident that the increased volatiles in both tea cultivars under drought stress exhibited a high degree of similarity, while the decreased VOCs showed some variations. Overall, the significantly increased volatiles predominantly displayed sweet, floral, earthy, fruity, and citrus aroma characteristics, whereas the decreased VOCs were mainly associated with fresh green, grassy, and leafy aromas.

Figure 7B provides the volcano plots for VOCs in the FD-D vs. FD-CK and WNZ-D vs. WNZ-CK groups as measured by HS-SPME-GC-MS, with increased and decreased VOCs highlighted in red and blue, respectively. A total of 55 VOCs were significantly elevated, and 47 VOCs were reduced in the drought-treated groups compared to the control groups for the FD and WNZ cultivars. Detailed information regarding these VOCs is available in Tables S6 and S7.

For the FD-D vs. FD-CK comparison, 30 VOCs were significantly elevated, including dodecanamide, dihydro-β-ionol, benzaldehyde, benzeneacetaldehyde, 2-hexenal, alkenes, medium- and long-chain fatty alcohols, and unsaturated fatty alcohols. In contrast, 25 VOCs were significantly reduced, primarily consisting of linalool oxide IV, linalool oxide III, coumarin, sesquiterpenes, and several green-odor-type alcohols and esters. In the WNZ-D vs. WNZ-CK comparison, 23 VOCs were significantly elevated and 24 VOCs were significantly reduced. The elevated VOCs mainly exhibited sweet, floral, and fruity odors, including volatile terpenoids (VTs), volatile phenylpropanoids/benzenoids (VPBs), and medium- to long-chain volatile fatty acid derivatives (VFADs, including fatty acid aldehydes, fatty acid alcohols, and fatty acid esters). Examples include benzeneacetaldehyde, 2,4,6-trimethyl-benzaldehyde, linalool oxide I, linalool oxide II, linalool oxide III, (3Z)-β-ocimene, α-farnesene, γ-muurolene, α-guaiene, methyl salicylate, (Z)-jasmone, and sulcatol. On the other hand, significantly downregulated VOCs mainly displayed citrus, woody, and herbal aromas, such as (E)-β-farnesene, D-limonene, linalool oxide IV, α-calacorene, δ-guaiene, cedrol, α-cubebene, δ-3-carene, as well as citrus/green-odor-type VFADs (fatty acid alcohols and fatty acid esters), such as non-(2E)-enol, cyclohexanol, cycloheptanol, (E)-3-hexen-1-ol, 1-octanol, (Z)-3-hexenyl hexanoate, (E)-butanoic acid 3-hexenyl ester, hex-(3Z)-enyl acetate, cis-3-hexenyl cis-3-hexenoate, and nonanal.

In comparison to HS-GC-IMS, HS-SPME-GC-MS detected a greater diversity of VOCs, with more pronounced up- and downregulation. The elevated VOCs detected by both methods mainly featured sweet, floral, and fruity characteristics, whereas the reduced VOCs were predominantly associated with green and herbal aromas. Notably, our findings on major differences in linalool oxides, jasmone, α-farnesene [23], methyl salicylate [18], 2-hexenal [49], alkenes, medium- and long-chain fatty alcohols, and unsaturated fatty alcohols [50,51,52] are consistent with previous reports. It is also noteworthy that methyl salicylate has been reported to induce stomatal closure in guard cells [53], which may aid in plant self-protection during drought stress.

Furthermore, compared to the control groups, the drought-stressed groups displayed a significant reduction in sesquiterpenes but an increase in monoterpenes, monoterpene oxides, and medium- to long-chain VFADs, particularly fatty alcohols. Similarly, drought has been reported to induce significant increases in alkanes and fatty alcohols in wheat leaves during both the seedling and flowering stages [54]. Numerous studies suggest that fatty alcohols may enhance drought resistance by modulating gene expression related to drought response, potentially through interactions with transcription factors. For instance, the OsFAR1 gene in rice has been implicated in primary fatty alcohol biosynthesis and plays a critical role in drought stress response [52]. Additionally, fatty alcohols act as organic solutes that help reduce cellular osmotic potential, thereby maintaining turgor pressure and supporting normal physiological processes under drought conditions. Long-chain VFADs, including fatty alcohols, have been shown to reduce water loss by forming cuticular waxes, thereby enhancing plant drought tolerance [50,51]. In conclusion, the accumulation of medium- and long-chain fatty alcohols, unsaturated fatty alcohols, and alkanes represents a key physiological adaptation to drought stress in plants, playing an essential role in osmotic regulation and the modulation of drought-responsive gene expression.

### 3.5. Analysis of Differential Metabolite KEGG Enrichment Pathways in Different Tea Cultivars Subjected to Drought Stress

**Figure 8:**
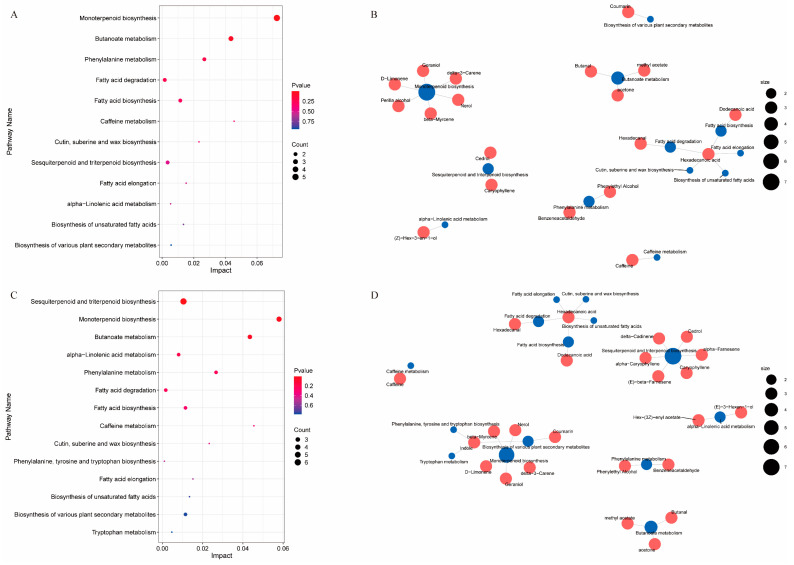
Pathway enrichment analysis of significantly altered VOCs of FD-D vs. FD-CK and WNZ-D vs. WNZ-CK. Differential VOC KEGG enrichment pathway bubble maps (**A**,**C**) and network maps of key target volatiles (**B**,**D**) of FD-D vs. FD-CK and WNZ-D vs. WNZ-CK, respectively.

To further elucidate the pivotal metabolic pathways associated with the alterations in VOCs for different tea cultivars subjected to drought stress, we performed a comprehensive VOC metabolite pathway enrichment analysis. This analysis enabled the visualization of pathway significance and weight through the enrichment of volatiles with analogous functions. As depicted in Figure 8, KEGG metabolic pathway analysis identified a total of 12 and 14 differential metabolic pathways for the comparisons of FD-D vs. FD-CK and WNZ-D vs. WNZ-CK, respectively. Comprehensive details regarding *p*-values, false discovery rates (FDRs), impact factors, compound names, and pathway links are provided in Tables S8 and S9. The identified pathways encompassed monoterpenoid biosynthesis, butanoate metabolism, phenylalanine metabolism, fatty acid degradation, fatty acid biosynthesis, caffeine metabolism, cutin, suberine, and wax biosynthesis, sesquiterpenoid and triterpenoid biosynthesis, fatty acid elongation, alpha-linolenic acid metabolism, the biosynthesis of unsaturated fatty acids, and the biosynthesis of various secondary metabolites. Notably, these 12 pathways were consistently observed in both FD and WNZ. In addition, the WNZ-D vs. WNZ-CK comparison revealed two supplementary enriched pathways: phenylalanine, tyrosine, and tryptophan biosynthesis, alongside tryptophan metabolism, primarily attributed to the differential accumulation of the target compound indole.

As depicted in Figure 8A,B, the pathways enriched in FD-D vs. FD-CK involved 19 differential VOCs across eight metabolic network pathways. The most significantly enriched metabolic pathway (*p* < 0.05) was monoterpenoid biosynthesis, which included six differentially accumulated volatiles: nerol, geraniol, perilla alcohol, beta-myrcene, D-limonene, and delta-3-carene. Remarkably, nerol and perilla alcohol levels increased in the FD-D group, whereas geraniol, beta-myrcene, D-limonene, and delta-3-carene were more abundant in the FD-CK group. Moreover, in the WNZ-D vs. WNZ-CK analysis, 23 differential VOCs were linked to seven metabolic networks. The pathways of sesquiterpenoid and triterpenoid biosynthesis, as well as monoterpenoid biosynthesis, were significantly enriched (*p* < 0.05). Within the sesquiterpenoid and triterpenoid biosynthesis pathway, the differentially accumulated VOCs included delta-cadinene, caryophyllene, alpha-farnesene, (E)-beta-farnesene, alpha-caryophyllene, and cedrol. In contrast, the enriched VOCs in the monoterpenoid biosynthesis pathway included nerol, geraniol, beta-myrcene, D-limonene, and delta-3-carene. Following drought stress, the only VOC exhibiting increased expression in the WNZ-D cultivar within these two enriched terpenoid metabolic pathways was alpha-farnesene, while the remaining enriched VOCs were found in higher concentrations in the WNZ-CK group. The findings suggest that drought stress markedly influences the accumulation of sesquiterpenes, resulting in a greater prevalence of terpene oxides, including linalool oxides and caryophyllene oxides.

In conclusion, both FD and WNZ cultivars were implicated in fatty acid degradation, fatty acid biosynthesis, fatty acid elongation, the biosynthesis of unsaturated fatty acids, and cutin, suberine, and wax biosynthesis post-drought stress. This indicates that tea plants may sustain cellular osmotic pressure and physiological functionality through the synthesis of medium-chain VFADs and unsaturated fatty acids, while simultaneously mitigating water loss via cutin, suberine, and wax biosynthesis to endure drought conditions [50,51,52,54]. Additionally, phenylalanine metabolism is closely linked to plant growth and environmental interactions [55]. Extensive research has demonstrated that numerous phenylpropanoids play critical roles in the response to drought stress [56,57,58]. Phenylpropanoids are vital aromatic compounds in tea leaves, significantly influencing quality formation and stress response [59]. Our study identified notable differences in the enrichment of benzeneacetaldehyde and phenylethyl alcohol within the phenylalanine metabolism pathway, particularly in the WNZ cultivar, where drought stress markedly elevated these compounds. This observation aligns with prior findings highlighting the significant increase in phenylethyl alcohol under drought conditions [18], contributing to enhanced rose floral aroma characteristics in tea leaves. 

Collectively, these results indicate that these metabolic pathways and their associated metabolites are crucial determinants influencing the VOC composition and aroma expression of tea leaves under drought stress.

## 4. Conclusions

By employing HS-GC-IMS and HS-SPME-GC-MS techniques alongside PLS-DA modeling, this study delineated significant alterations in the VOCs of FD and WNZ tea cultivars subjected to drought stress. The drought-stressed groups exhibited a marked increase in both the concentration and diversity of VOCs associated with sweet, fruity, caramel-like, floral, and earthy notes, while green, grassy, leafy, woody, herbal, and floral aromas were diminished. Notably, there was an elevated presence of methylated compounds, linalool oxides, and medium- to long-chain VFADs in the drought-treated samples. Conversely, sesquiterpenoid content and diversity were substantially reduced. The varietal responses to drought stress were distinct, with FD-D showing an increased diversity of VOCs, whereas WNZ exhibited a greater increase in the concentrations of low-threshold VOCs.

Among the four sample groups, key aroma-active compounds (OAV > 1) included 1-Octen-3-ol, linalool, geraniol, β-damascone, (E)-β-ionone, citral, and β-myrcene, with linalool exhibiting the highest OAV, underscoring its role as a primary aroma contributor in both tea cultivars. Based on VIP > 1 and OAV > 1 thresholds, 8 and 13 potential key aroma markers were identified for distinguishing between the different cultivars and treatments using the PLS-DA model for HS-GC-IMS and HS-SPME-GC-MS, respectively. Additionally, KEGG enrichment analysis revealed significant alterations in 12 metabolic pathways following drought stress. The sesquiterpenoid and triterpenoid biosynthesis pathways, as well as monoterpenoid biosynthesis, exhibited significant changes (*p* < 0.05). Furthermore, pathways related to fatty acid synthesis, elongation, degradation, the biosynthesis of unsaturated fatty acids, linalool oxides, cutin, suberine and wax biosynthesis, and phenylalanine metabolism were also enriched, further validating the observed increases in the abundance and diversity of linalool oxides and medium- to long-chain VFADs, along with the pronounced reduction in sesquiterpenoid compounds under drought stress. 

Overall, these results provide a comprehensive insight into how drought stress affects VOC accumulation in fresh tea leaves across different cultivars, while highlighting the corresponding alterations in key metabolic pathways. This knowledge establishes a foundation for optimizing water management strategies in tea plant cultivation, aiming to enhance flavor quality. The current study relies on metabolomics and flavoromics to analyze key volatile aroma compounds and their metabolic pathways in tea leaves subjected to 20 days of continuous field drought stress. However, the absence of physiological or genetic data linking VOC changes to drought resistance presents a limitation. Future research will aim to integrate physiology, transcriptomics, and DNA methylation data to elucidate the molecular mechanisms governing VOC accumulation and aroma formation in tea under drought stress.

## Figures and Tables

**Table 1:** The main aroma-active compounds with an odor activity value (OAV) ≥ 0.1 in 4 groups.

NO.	Volatile Compounds	Odor Characteristics	OT (μg/L)	OAVs			
				FD-CK	FD-D	WNZ-CK	WNZ-D
1	Hex-(3Z)-enyl acetate	Green, fruity	13	0.19	-	1.58	0.25
2	Methyl salicylate	Minty, green	40	0.12	0.17	0.27	1.18
3	1-Hexanol	Herbal, fruity	5.6	0.62	0.63	0.64	0.64
4	1-Heptanol	Green, leafy, violet	5.4	0.68	0.79	0.7	0.7
5	1-Octen-3-ol	Earthy, mushroom	1.5	2.36	2.53	2.35	2.76
6	(Z)-Hex-3-en-1-ol	Green, grassy	3.9	1.66	-	-	-
7	1-Octanol	Waxy, green, orange	125.8	0.03	0.06	0.1	-
8	Linalool	Floral, citrus, rose	0.22	3155.46	1577.42	2307.87	2190.92
9	1-Nonanol	Floral, rose, orange	45.5	-	-	0.16	-
10	Geraniol	Floral, rose	1.1	119.45	115.99	130.9	99.64
11	6-methyl-5-Hepten-2-one	Citrus, lemon	0.16	-	1.68	-	3.06
12	1-Octen-3-one	Earthy, herbal, mushroom	0.003	-	-	-	113.76
13	(Z)-Jasmone	Floral, woody, herbal	0.26	9.31	0.16	-	1.81
14	(E)-alpha-Ionone	Violet	0.1	-	5.09	2.76	29.44
15	beta-Damascone	Fruity, floral, berry	0.002	9.32	19.18	60.44	157.58
16	(E)-beta-Ionone	Foral, fruity, woody	0.007	217.88	8.6	746.08	658.21
17	Hexanal	Green, grassy	5	0.61	1.34	0.81	1.24
18	2-Hexenal	Green, almond, fruity	30	0.16	0.66	0.14	0.67
19	Heptanal	Green, fatty, herbal	2.8	0.71	0.7	-	0.68
20	(E)-2-Heptenal	Green, vegetable, fatty	13	-	0.14	-	0.16
21	(E,E)-2,4-Heptadienal	Fatty, green	0.032	-	58.5	-	59.41
22	Octanal	Aldehydic, citrus	0.587	4.02	4.45	-	4.13
23	Oct-(2E)-enal	Fatty, green, herbal	0.2	-	-	-	9.83
24	Benzeneacetaldehyde	Green, floral, hyacinth	5.2	-	0.19	-	0.2
25	Nonanal	Aldehydic, orange, rose	1.1	2.79	3.18	2.78	-
26	(E)-2-Nonenal	Fatty, cucumber, citrus	0.19	10.01	-	-	10.78
27	Decanal	Aldehydic	3	0.74	0.96	0.71	0.91
28	(E)-2-Decenal	Fatty, earthy, green	0.3	7.9	7.82	7.48	10.78
29	Nona-(2E,4E)-dienal	Fatty, melon, green	0.1	-	-	-	19.16
30	Neral	Citrus, lemon	53	0.04	0.04	0.62	-
31	Citral	Citrus, lemon, sweet	40	1.06	1.43	1.42	1.43
32	Undecanal	Aldehydic, floral, citrus	12.5	0.16	-	-	-
33	Dodecanal	Aldehydic, floral, citrus	0.13	-	16.5	-	-
34	Tridecanal	Aldehydic, citrus, grapefruit	10	-	0.19	-	-
35	beta-Myrcene	Spicy, peppery, woody	1.2	3.85	2.83	3.85	3.1
36	D-Limonene	Citrus, orange	34	0.1	0.09	0.1	-
37	(E)-beta-Ocimene	Herbal, citrus, woody	34	0.13	0.09	0.11	0.16
38	alpha-Farnesene	Woody, citrus, herbal	87	-	-	-	0.24
39	delta-Cadinene	Woody, spicy, burnt	1.5	2.36	-	3.38	2.4
40	Cedrol	Woody, cedarwood	0.5	6.54	6.03	5.75	-
41	Linalool oxide I	Woody, flowery, earthy	100	0.98	1.4	1.3	6.41
42	Linalool oxide II	Woody, flowery	190	0.76	0.95	0.36	5.72
43	Coumarin	Tonka	25	0.14	-	0.15	0.17
44	Naphthalene	Pungent, dry resinous	6	0.59	0.6	0.58	0.59
45	Indole	Flowery	40	-	-	0.13	0.13

Notes: odor characteristics and odor threshold (OT) were referenced from sources denoted as in Table S4.

## Data Availability

The original contributions presented in this study are included in the article/Supplementary Materials; further inquiries can be directed to the corresponding authors.

## Supplementary Materials

The following supporting information can be downloaded at: https://www.mdpi.com/article/10.3390/foods13213412/s1, Figure S1. Close-up view of a PCA biplot displaying VOCs concentrations detected by HS-SPME-GC–MS.; Table S1: Composition of model stock solution for volatile-free tea infusion blank matrix; Table S2: Calibration standards curves of the key VOCs; Table S3: The information of volatile compounds identified by HS-GC-IMS; Table S4: Identification and quantification of the VOCs, odor description, and OAV analysis in this study by HS-SPME-GC–MS; Table S5: Importance features within PLS-DA using VIP values for HS-SPME-GC-MS data; Table S6: Significant change profiles in the accumulation of VOCs for FD-D vs. FD-CK based on HS-SPME-GC-MS data; Table S7: Significant change profiles in the accumulation of VOCs for WNZ-D vs. WNZ-CK based on HS-SPME-GC-MS data; Table S8: Detailed information on KEGG-enriched metabolic pathways for FD-CK and FD-D with reference to *Arabidopsis*; Table S9: Detailed information on KEGG-enriched metabolic pathways for WNZ-CK and WNZ-D with reference to *Arabidopsis.* References [60,61,62,63,64,65,66,67,68] are cited in the supplementary materials
