# Supplementary figures and images for: Impact of Mild Field Drought on the Aroma Profile and Metabolic Pathways of Fresh Tea (Camellia sinensis) Leaves Using HS-GC-IMS and HS-SPME-GC-MS
Source: Foods. 2024 Oct 26;13(21):3412. doi: 10.3390/foods13213412 (PMC11544960; doi:10.3390/foods13213412)

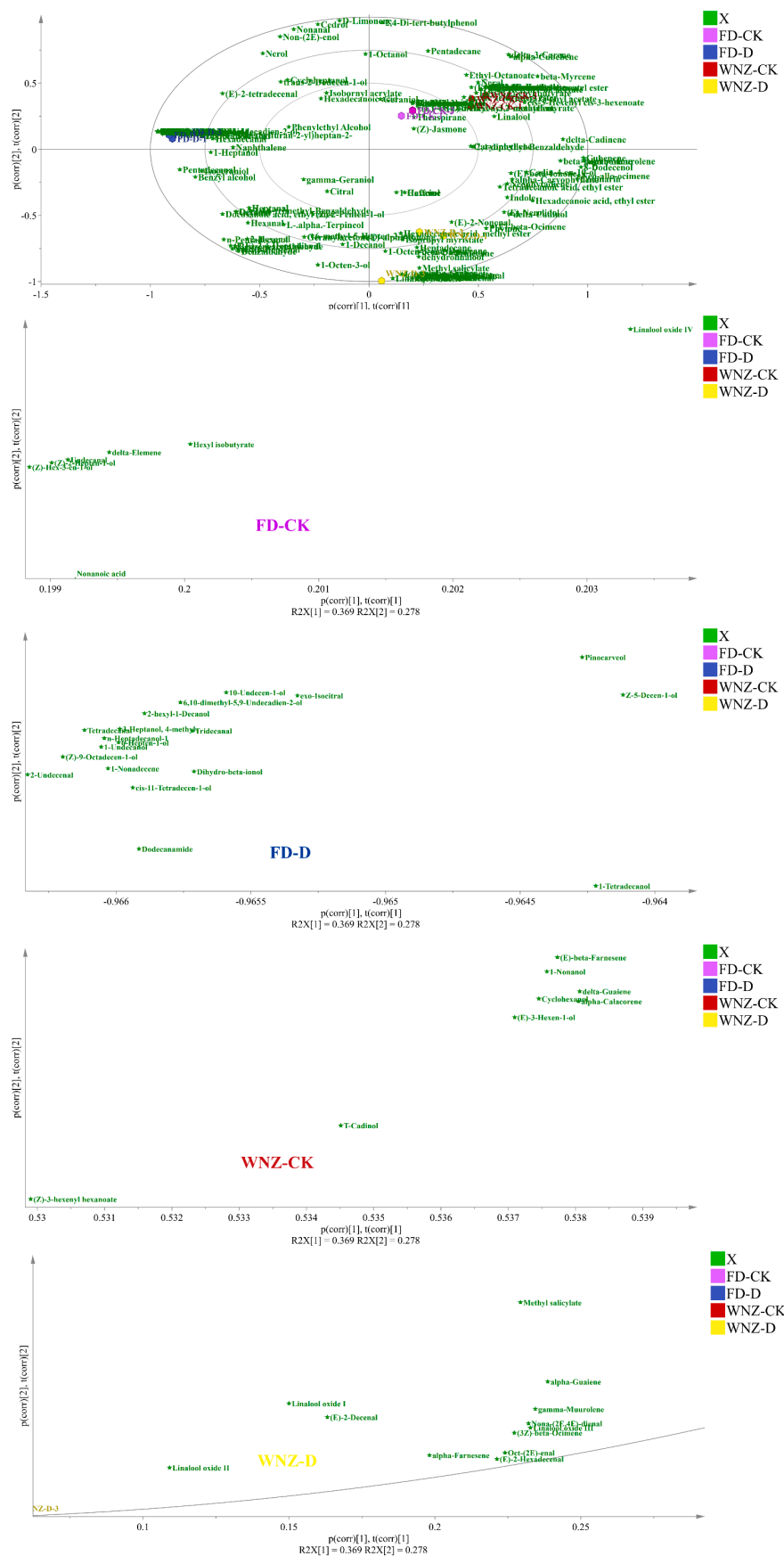

**Figure S1.** Close-up view of a PCA biplot displaying VOCs concentrations detected by HS-SPME-GC-MS.

Supplement: Supplementary file 1 [file foods-13-03412-s001.zip › Supplementary Materials-Figure S1.pdf]
